# Harnessing belowground processes for sustainable intensification of agricultural systems

**DOI:** 10.1007/s11104-022-05508-z

**Published:** 2022-06-22

**Authors:** Eva Oburger, Hannes Schmidt, Christiana Staudinger

**Affiliations:** 1 Department of Forest and Soil Science, Institute of Soil Research, University of Natural Resources and Life Sciences, Konrad Lorenzstrasse 24, 3430 Tulln an der Donau, Austria; 2 Centre for Microbiology and Environmental Systems Science, University of Vienna, Djerassiplatz 1, 1030 Vienna, Austria; 3 Graduate School of Integrated Sciences for Life, Hiroshima University, Kagamiyama 1-7-1, Higashi-Hiroshima, Japan

**Keywords:** Soil structure, Water availability, Root exudation, C cycling, C sequestration, Plant nutrition, Plant health, Soil health, Root exudation, Plant-plant interaction, Microbes, Rhizobiome, Intercropping

## Abstract

Increasing food demand coupled with climate change pose a great challenge to agricultural systems. In this review we summarize recent advances in our knowledge of how plants, together with their associated microbiota, shape rhizosphere processes. We address (molecular) mechanisms operating at the plant–microbe-soil interface and aim to link this knowledge with actual and potential avenues for intensifying agricultural systems, while at the same time reducing irrigation water, fertilizer inputs and pesticide use. Combining in-depth knowledge about above and belowground plant traits will not only significantly advance our mechanistic understanding of involved processes but also allow for more informed decisions regarding agricultural practices and plant breeding. Including belowground plant-soil-microbe interactions in our breeding efforts will help to select crops resilient to abiotic and biotic environmental stresses and ultimately enable us to produce sufficient food in a more sustainable agriculture in the upcoming decades.

## Introduction

The increasing availability of industrial fertilizer and pesticides combined with the implementation of high yield plant varieties sparked the first Green Revolution in the middle of the twentieth century resulting in a massive increase in cereal yield worldwide. At that time, plant breeders focused on aboveground plant features, developing crops that would produce high yields under plentiful water and nutrient supply. While highly successful in parts of the world, with staple food yields doubling or even tripling, the Green Revolution brought little change to the areas worst affected by hunger and malnutrition (Lynch 2019). In addition to unaffordable fertilizers, soils in low-income countries are often affected by limited nutrient availability and yields regularly suffer from drought, diseases and herbivory (Lesk et al. 2016; Lynch 2007; Ristaino et al. 2021; White and Broadley 2009). To meet the growing food demand, the next Green Revolution will have to focus on improving yields on infertile soils with minimal fertilizer inputs (Lynch 2007). Furthermore, changing climatic conditions and corresponding ecosystem responses raise the need for crops tolerant to various environmental stressors including drought, salinity as well as pathogen infection.

**Fig. 1:**
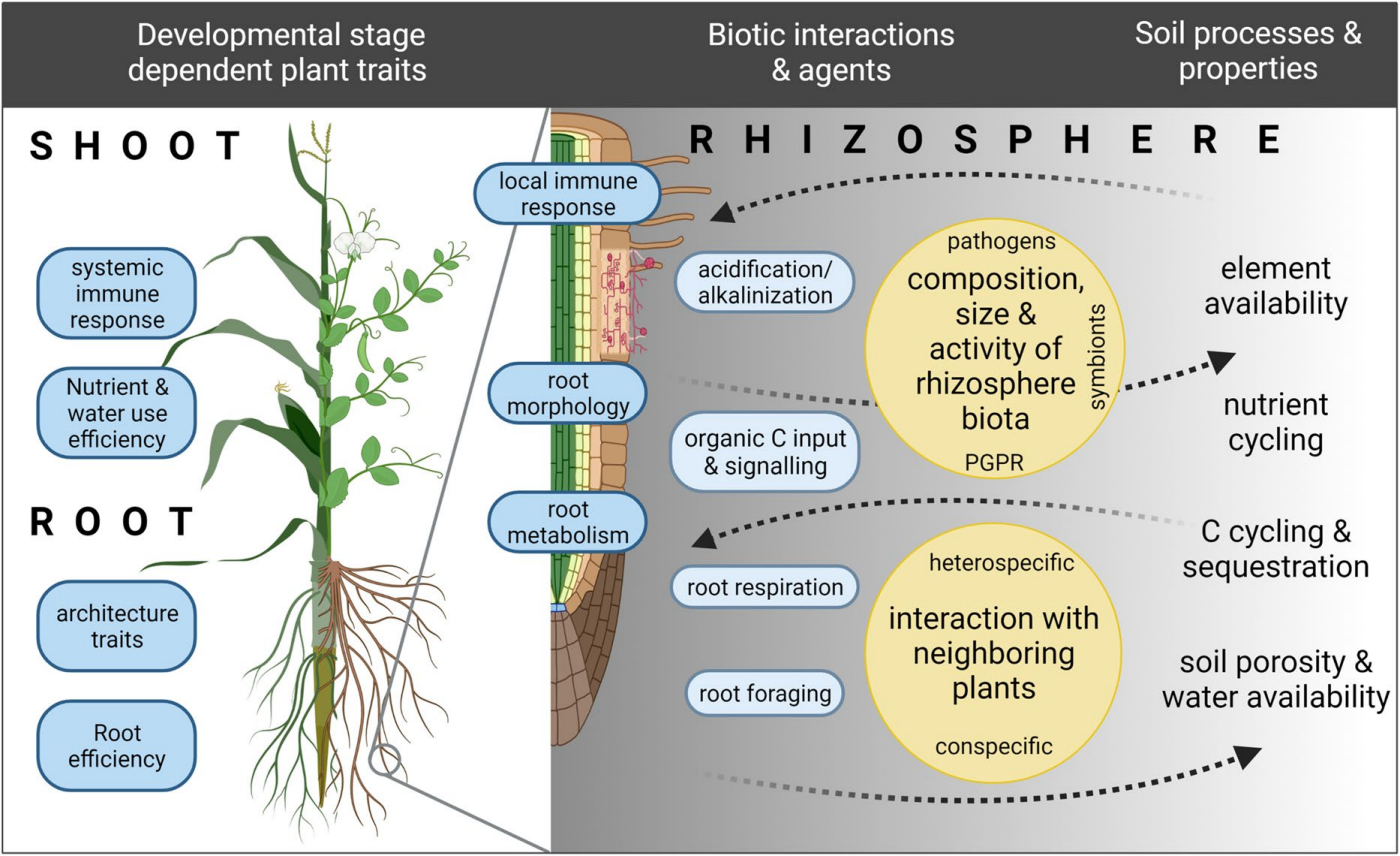
Summary figure of plant traits, biotic agents and interactions shaping rhizosphere soil properties and processes and vice versa. Root efficiency is defined as mass unit nutrient or water taken up per unit root surface area. Created with biorender.com

Plant species (and even varieties) are known to differ in their root resource acquisition efficiency (which we here define as mass unit nutrient or water taken up per unit root surface area) (Mori et al. 2016) as well as in their internal water/nutrient use efficiency (Chochois et al. 2015; St Aime et al. 2021; Tron et al. 2015) and their level of tolerance against biotic and abiotic stressors (Al-Tamimi et al. 2016; Gioia et al. 2015; Oladzad et al. 2019). These genetically determined differences are driven by the plant phenotype which integrates root architectural and morphological traits, as well as general plant metabolism including systemic and local immune responses (summary Fig. 1). In addition, these species- and genotype-specific traits can change with plant development. It is well established, that growth and metabolic activity of plant roots can alter the physicochemical properties and the biological activity in the soil surrounding roots (i.e. the rhizosphere) and therefore, in turn, significantly affect plant growth performance. Rhizosphere properties emerging from plant–microbe-soil interactions are of crucial importance as they ultimately determine the plants’ nutrient and water availability and impact pathogen infection as well as the establishment of symbiotic relationships (Lambers et al. 2009)(see also Fig. 1). Living plants interact with the soil matrix not only by taking up water and nutrients but also by actively and passively releasing inorganic (H_3_O^+^, CO_2_, O_2_) and organic compounds (i.e. photosynthates and derivatives, controlled release of root border cells as well as cell debris and sloughed-off root cap cells) into the soil (Oburger and Jones 2018). The zone of influence (i.e. rhizosphere extent) depends on the process and varies dynamically through space and time; however, the spatial extent of the rhizosphere typically does not exceed a few mm (e.g. Hinsinger et al. 2009; Kuzyakov and Razavi 2019). Even though rhizosphere processes occur at a small scale, they shape agricultural productivity and influence biogeochemical element cycling and soil development and are consequently of global importance (Finzi et al. 2015). Together with the soil biota, roots (re-) organize particle aggregation, soil pore volume and soil pore connectivity and are therefore affecting the global water cycle by altering water infiltration, storage and aeration (Bengough 2012). In addition, the continuous input of organic carbon (C) by roots into the soil alters size, composition and activity of the rhizosphere microbiome and consequently drives a wide range of processes and feedback loops in the rhizosphere affecting plant growth and plant nutrition (Hayat et al. 2010; Lugtenberg and Kamilova 2009) as well as ecosystem response to climate change (Classen et al. 2015; Langley and Hungate 2014). Roots therefore play a central role in C cycling and C sequestration (Dijkstra et al. 2021). Mediated by the soil biota, roots also trigger the solubilization and redistribution of nutrients other than C and N between organic and inorganic pools as they explore and exploit the soil (Jones and Oburger 2011; Oburger et al. 2011; Vetterlein et al. 2020).

Understanding and harnessing plant traits and related rhizosphere processes involved in improved crop and soil health is considered a key strategy to sustainably intensify agricultural systems and therefore increase food and fodder production (de la Fuente Cantó et al. 2020; Lynch 2019; Staudinger et al. 2016). In the past decades, intensive research combined with methodological development allowed us to gain considerable mechanistic insights into individual rhizosphere processes (Baveye et al. 2018; Oburger and Schmidt 2016; Schnepf et al. 2022). However, the focus was mostly on individual mechanisms rather than on the interactions of several processes co-occurring in the rhizosphere. Undoubtedly, these studies significantly advanced our knowledge about the plant–microbe-soil system. However, it is increasingly recognized that a holistic view of occurring processes and their feedback loops is needed to further our understanding of the complex interplay of processes giving rise to desirable rhizosphere properties (Vetterlein et al. 2020). Ultimately this knowledge should help us to manage soils and crop growth in a more sustainable and efficient way in the future. In this review, we aim to summarize recent advances in our understanding of how plants shape rhizosphere processes and discuss approaches that have already been applied in agroecosystems or showed promising results in vitro. Keeping the plant perspective in focus of this review, we fully acknowledge the ‘holobiont concept’ and will discuss plant-(micro)biota interactions that may lead to an ‘extended phenotype’ in the respective sections.

## Soil structure and water availability

It is well known that increases in soil bulk density do not only affect the soil’s water infiltration capacity but also result in changes in root morphology and root system architecture, with higher bulk densities generally resulting in thicker, shorter roots than roots grown in soils with lower bulk density (Correa et al. 2019; Pandey et al. 2021). However, growing roots themselves can also locally alter soil porosity and soil aggregation affecting aeration, water infiltration as well as saturated and unsaturated soil water flow. In turn, this can have an impact on plant growth performance as well as on microbial abundance and activity and related biogeochemical cycles. Growing roots were found to displace soil particles and to locally increase bulk density in their close vicinity (Aravena et al. 2011; Bruand et al. 1996). Contrastingly, several X-ray computed tomography-based 3D imaging studies also revealed the opposite effect reporting an increase in soil porosity in the rhizosphere with densification only being found (if at all) at some distance away from the roots (Helliwell et al. 2019). Carminati et al. (2013) and Koebernick et al. (2019) made similar observations and attributed the higher rhizosphere porosity to gap formation and larger pore diameters caused by loose packing between the convex root surface and convex soil particles. In a recent study, Lucas et al. (2019) aimed to reconcile these contrasting findings and demonstrated that soil compaction in the rhizosphere only occurred when macroporosity was low and dominated by isolated pores. However, the authors also observed a more porous rhizosphere compared to the bulk soil when roots were grown in soils characterized by a highly connected macropore system. The authors concluded that growth-driven rhizosphere compaction only occurs if the initial soil structure does not offer sufficient volume of well-connected macropores. Another recent study indicates that the magnitude of bulk density alteration in vicinity of roots is dependent on the underlying soil texture and structural heterogeneity (Phalempin et al. 2021) which highlights the importance of recognizing interactions and the extent of their effects between soil structure and root traits as a two-way system (i.e. changes in soil structure due to plants and changes in root growth due to soil structure).

Higher porosity in the rhizosphere will improve air permeability and increase water infiltration and saturated water flow. On the other hand, root water uptake could be negatively affected under non-saturated or drying conditions (Aravena et al. 2011). Research in the past decade revealed increasing evidence that mucilage released by the tip of growing roots or root hairs can alter rhizosphere soil physical and hydraulic properties, maintaining the connectivity of the liquid phase in increasingly dry conditions. Mucilage is a polymeric gel primarily composed of neutral and acid polysaccharides that is mainly released from root cap cells at the root tip (Carminati and Vetterlein 2013). Current evidence suggests that mucilage has a lower surface tension and a higher viscosity than water thereby preventing the breakup of the liquid phase during drying and maintaining the physical connection between the soil matrix and the root surface (Carminati et al. 2017, 2013). Together with root hairs, mucilage is considered to be an important driver of soil particle aggregation and rhizosheath formation (i.e. layer of soil adhering to the root surface)(Galloway et al. 2018) which is expected to maintain physical contact between soil and roots upon soil drying (Bengough 2012). While mucilage has been shown to keep the rhizosphere wetter than the bulk soil during initial soil drying, it turned hydrophobic after severe drought causing initial water repellence in the rhizosphere upon rewetting. Changing hydraulic properties of soil not only influences its physico-chemical properties but most likely also soil (micro-)biota, especially under conditions of decreasing water availability. Increasing the connectivity between soil and roots was shown to improve the diffusion of nutrients in the soil aqueous phase during soil drying (Zarebanadkouki et al. 2019). Furthermore, root mucilage may help to create heterogeneous niches for microbial growth and interactions through reduced fluid flow relative to the soil solution (Nazari et al. 2022; Stewart 2003). A limitation of diffusion likely results in the accumulation of plant low-molecular weight compounds, which are preferentially released in apical root zones in the mucilage layer, favoring chemoattraction, exchange of signaling molecules and defense compounds, as discussed for microbial biofilms (Flemming et al. 2016).

Besides representing an energy-rich substrate and sustaining aqueous phases in dry soils, it was recently hypothesized that root mucilage containing also plant-derived proteins and extra cellular DNA could further provide the first line of defense against plant-pathogens (Driouich et al. 2021; Staudinger et al. 2022). To date, results from a limited number of studies revealed plant species-specific and even root type-dependent differences in mucilage composition and properties (Naveed et al. 2019; Zickenrott et al. 2016). The chemical composition of mucilage released from plant roots shares similarities with the composition found in primary plant cell walls, as the major structural components are pectic polysaccharides and glycoproteins including arabinogalactans and extensins (Bacic et al. 1986; Driouich et al. 2013; Staudinger et al. 2021). Although new methods of pectin detection have been developed recently (e.g. Anderson et al. 2012), considerable knowledge gaps exist with regards to pectin biosynthesis, intracellular trafficking and secretion (Anderson 2016). According to a widely held view, mucilage is mainly secreted from root hair tips and the root apical region and together with secretions of microbial origin, a thin layer of mucigel can be formed around young root sections (McCully 1995). Due to the difficulties of sampling mucilage in natural soil growth conditions (Oburger and Jones 2018), the implications of these differences in plant water as well as nutrient uptake consequently remain unknown so far (Vetterlein et al. 2020). Combining our current knowledge about the two-way interaction of soil structure and root morphological development with an in-depth understanding of species-specific mucilage properties and related functions in maintaining water connectivity in the rhizosphere could significantly help our efforts to improve drought tolerance in crops. This might be of particular relevance for deep rooting varieties, as it has been shown that deep rooting genotypes are generally more drought tolerant than shallow rooting ones (Lynch 2007).

## Root exudates – a key to understanding rhizosphere processes

Next to their effect on soil physical properties, roots release a large diversity of soluble or volatile organic molecules (i.e. root exudates) as well as cell debris and sloughed-off root cap cells (which all together make up rhizodeposition) as they forage for water and nutrients. These root exudates (and other rhizodeposits) play a central role in rhizosphere processes as they spark a cascade of feedback loops between roots, the associated microbiome and soil particles. Release mechanisms and functional importance of root exudates, particularly regarding nutrient mobilization and cycling as well as the interaction with microorganisms have already been discussed in numerous reviews to which we refer our reader for further details (e.g. Badri and Vivanco 2009; Canarini et al. 2019; Coskun et al. 2017; Dennis et al. 2010; Hacquard et al. 2017; Sasse et al. 2018; Vives-Peris et al. 2020).

Besides triggering physicochemical processes such as mineral weathering, soil aggregation, and nutrient mobilization, it is well acknowledged that exudates act as signaling compounds between plants and microbiota and that exudate quality and quantity shape the rhizosphere microbiome (Reinhold-Hurek et al. 2015; Sasse et al. 2018). Furthermore, it has been shown that particularly under pathogen attack exudation is altered to recruit beneficial microbes that in turn trigger induced systemic resistance responses (i.e. systemic activation of plant defenses by hormone signaling upon pathogen attack) in the plant (Berendsen et al. 2018; Rudrappa et al. 2008; Yuan et al. 2018; Zhang et al. 2020). However, many underlying mechanistic details are still poorly understood, mainly due to our lack of knowledge on compound identity and diversity exuded from different species as well as under different environmental conditions. Thanks to recent developments not only in analytical instrumentation, but also in computing power, available data processing software as well as metabolite databases, the number of non-targeted metabolomic exudation studies aiming to reveal the entire metabolite composition released by roots has significantly increased in the past five years. These analytical advances allowed for better insights into how root exudates change with plant development (Zhalnina et al. 2018) as well as upon altered environmental conditions including nutrient availability (Smercina et al. 2021; Tantriani et al. 2020; Wang et al. 2022; Ziegler et al. 2016), soil pollution (Frémont et al. 2022; Wang et al. 2021a), drought (Gargallo-Garriga et al. 2018; Ghatak et al. 2022), pathogen infection (Balendres et al. 2016; Zhang et al. 2020), inoculation with symbionts and beneficial rhizobacteria (Riviezzi et al. 2021) and intercropping (Vora et al. 2021), as well as on how exudation differs between different genotypes (Lopez-Guerrero et al. 2022; Mönchgesang et al. 2016). Furthermore, combining in-depth exudate analysis with improved microbiome profiling techniques also led to significant progress regarding our knowledge of effects of specific exudate compounds or compound classes on the soil microbiome and/or other rhizosphere processes in the past decade. Table 1 provides an overview of specific mechanisms driven or influenced by individual root exudate compounds or compound classes in the rhizosphere that have been identified to date. Despite these recent advances, we are still far from deciphering the entire metabolite diversity exuded by plants and their function in the rhizosphere. Number and chemical nature of metabolites or features detected very much depend on the analytical approach applied (Escolà Casas and Matamoros 2021). Furthermore, available data bases used for compound identification to date only allow to identify about 10–30% of analyzed features (e.g. Frémont et al. 2022; Herz et al. 2018; van Dam and Bouwmeester 2016). While it is admittedly difficult to discuss unidentified metabolites, our interpretations and conclusions particularly in the context of plant–microbe interactions might still be prone to biases if we keep our sole focus on exudate metabolites that we can identify.

**Table 1:** Overview of identified mechanisms triggered/influenced by individual root exudate compounds or compound classes including the plant biosynthetic origin of precursors for exudate production, the plant species for which exudate release has been reported and (if known) the type of transporter or mechanism responsible for exudation. Metabolites are categorized based on the following main mechanisms: (a) establishment of symbiosis, (b) pathogen interaction & toxicity response, (c) nutrient availability (direct & indirect) & plant growth, (d) drought stress & soil structure, (e) microbial community composition in the rhizosphere, (f) plant-plant interaction. Note that individual root exudates can have several functions and are sometimes listed in multiple categories

**Compound class:** exudate compound	**Metabolic pathway**	**Mechanism**	**Release transporter/ mechanism**	**Exudation reported for**	**Reference**
**(a) Establishment of symbiosis**					
**Carotenoid—derivatives (Strigolactones):** Strigol, solanacol, sorgomol, orobanchol, sorgolactone, 4-deoxyorobanchol, 5-deoxystrigol	Methylerythritol phosphate (MEP) pathway, carotenoid biosynthesis	Triggering of mycorrhizal infection	ABC-type	Presumably all species forming mycorrhizal associations	Kretzschmar et al. (2012) Floková et al. (2020)
**Flavonoids—Flavone:** Rahmnetin, apigenin, quercetin, luteolin, hyperoside, rutin, myricetin, kaempferol, galangin	Phenylpropanoid biosynthesis & glycolysis	Stimulation of host penetration, hyphal growth or spore germination	ABCG-type? MATE? ^1^	Numerous species forming mycorrhizal symbiosis with *Glomus* (AM)*, Gigaspora* (AM)*, Suillus bovinus* (EM)	Cesco et al. (2012 and references therin)
**Flavonoids—Flavanones:** Hesperetin, naringenin	Phenylpropanoid biosynthesis & glycolysis	Stimulation of spore germination			
**Flavonoids—Isoflavonoids:** Daidzein, genistein	Phenylpropanoid biosynthesis & glycolysis	Stimulation of mycorrhizal colonization & spore germination			
**Flavonoids:** Izoliquiritigenin, liquiritigenin, daidzein, formomonetin, apigenin, afrormosin, medicarpin, vestitone	Phenylpropanoid biosynthesis & glycolysis	Triggering of root infection by *rhizobial* strains & nodule formation^4^	ABCG-type	Legumes, *Medicaco truncatula*	Banasiak et al. (2013)
**Flavonoids—Isoflavonoids:** Genistein	Phenylpropanoid biosynthesis & glycolysis	Triggering nodule infection Induction of fungal sporulation leading to vegetative growth reducing exudate consumption	LaMATE2	*Lupinus albus*	Biała-Leonhard et al. (2021); Weisskopf et al. (2006); Zhou et al. (2021)
**Terpenoids—Diterpene:** Abietic acid	MEP pathway	Stimulation of spore germination	Unknown	*Pinus sylvestris*	Fries et al. (1987)
**(b) Pathogen interaction & toxicity response**					
**Benzoxazinoids (BX):** 2,4-Dihydroxy-7-methoxy-1,4- benzoxazin-3-one glucose **(**DIMBOA-Glc), DIMBOA, N–O-methylated DIMBOA-Glc (HDMBOA-Glc)	Amino acid metabolism, tryptophan biosynthesis, indole metabolism	Inhibition of host recognition and virulence of pathogenic *Agrobacterium tumefaciens16*	Unknown^2^	*Zea mays*	Maresh et al. (2006)
**BX-degradation products:** 6-Methoxy-2-benzoxazolinone (MBOA), 2-benzoxazolinone (BOA)	Amino acid metabolism, tryptophan biosynthesis, indole metabolism	Inhibition of radial growth in 18 out of 29 *Fusarium* spp. tested, detoxification of MBOA and BOA in some *Fusarium* spp.	Unknown^2^	*Zea mays*	Glenn et al. (2001)
**Benzoxazinoids (BX):** DIMBOA-Glc, DIMBOA, HDMBOA-Glc	Amino acid metabolism, tryptophan biosynthesis, indole metabolism	Protection against general herbivores, however BX-Fe complexes mediated infection by Western corn root worm	Unknown^2^	*Zea mays*	Hu et al. (2018)
**Coumarins:** Scopoletin	Phenylpropanoid biosynthesis	Inhibition of soil-borne fungal pathogens *Fusarium* *oxysporum *and *Verticillium dahliae*	ABCG	*Arabidopsis thaliana*	Stringlis et al. (2018); Ziegler et al. (2017)
**Diterpene:** Rhizathalene A (semi-volatile)	MEP pathway	Improved defence upon insect herbivory	MATE? ^1^	*Arabidopsis thaliana*	Vaughan et al. (2013)
**Diterpenoids:** Dolabraxelin, kauralexin	MEP pathway	Antifungal bioactivity, modification of rhizosphere bacterial community	MATE? ^1^	*Zea mays*	Murphy et al. (2021)
**Glucosinolates:** Isothiocyanates	Amino acid metabolism	Toxic effect on soil-borne pathogens	MATE? ^1^	*Arabidopsis thaliana*	Bednarek et al. (2009); Bressan et al. (2009)
**Glycolipid:** Short-chained ascarosides (ascr#9)	Nematode origin: β-oxidation in plant peroxisomes of nematode secreted long-chained ascarosides	Repellence of parasitic nematodes	Unknown	*Arabidopsis thaliana, Solanum tuberosum*	Manohar et al. (2020)
**Mucilage:** Mix of polysaccharides, lipids, proteins	Amino acid metabolism, fatty acid metabolism, central C metabolism	Contains proteins with antimicrobial functions, protection from Al^3+^ toxicity via the formation of ionic bonds	Exocytosis	*Heliophila coronopifolia, Glycine max*	Cai et al. (2013); Morre et al. (1967); Weiller et al. (2016)
**Organic acid anions:** Citrate	Central carbon (C) metabolism, TCA cycle	Detoxification of Al^3+^ via complexation in the soil solution	MATE	*Hordeum vulgare*	Furukawa et al. (2007)
**Organic acid anions:** Malate	Central C metabolism, TCA cycle	Pathogen (*Pseudomonas syringae*) defence via attracting beneficial rhizobacterium *Bacillus subtilis* FB17 that induces biofilm formation	ALMT1^1^	*Arabidopsis thaliana*	Rudrappa et al. (2008)
**Organic acid anions:** Ferulate, tartarate, laurate, salicylate	Phenylpropanoid biosynthesis, Ascorbic acid metabolism, (and others)	Strong inhibitory effect on *P*h*ytophthora nicotianae* mycelium growth	unknown	*Nicotiana tabacum*	Zhang et al. (2020)
**Proteins:** β-1,3-Glucanases, chitinases, lipid transfer proteins (LTPs)	Amino acid metabolism	Inhibitory effect on growth of fungus *Fusarium oxysporum* in vitro	MDR (ABC)? ^1^	*Vigna unguiculata*	Nóbrega et al. (2005)
**(c) Nutrient availability (direct & indirect) & plant growth**					
**Benzoquinone:** Sorgoleone	Fatty acid metabolism & amino acid metabolism (methionine)	Biological nitrification inhibition (BNI) , suppression of plant growth	Exocytosis	*Sorghum bicolor*	Dayan et al. (2010); Subbarao et al. (2015)
**Benzoxaxinoids (BX):** DIMBOA-Glc, DIMBOA, HDMBOA-Glc	Amino acid metabolism, tryptophan biosynthesis, indole metabolism	Formation of BX-Fe complexes and improved Fe acquisition	IRT 1 (uptake)	*Zea mays*	Hu et al. (2018)
**Benzoxazinoids:** DIMBOA	Amino acid metabolism – tryptophan biosynthesis – indole metabolism	Triggering colonization of plant growth promoting bacterium *Pseudomonas putida*	Unknown^2^	*Zea mays*	Neal et al. (2012)
**Coumarins:** Scopoletin, scopolin, fraxetin, esculetin, esculin	Phenylpropanoid biosynthesis	Mobilization of Fe in strategy I species	ABCG	*Arabidopsis thaliana* *Brassica napus L., Raphanus sativus L., Sinapis alba L*	Sarashgi et al. (2021); Schmid et al. (2014)
**Diol:** 1,9-Decanediol	Central C metabolism	Biological nitrification inhibition, correlation to N use efficiency	MATE? ^1^	*Oryza sativa*	Sun et al. (2016)
**Diterpene:** Brachialactone	MEP pathway	Biological nitrification inhibition	MATE? ^1^	*Brachiaria humidicola*	Subbarao et al. (2009)
**Non-proteinogenic amino acids—Phytosiderophores:** 2’-Deoxymugineic acid, 3-*epi*-hydroxy-2’-deoxymugineic acid, hydroxy-2’-deoxymugineic acid, mugineic acid, 3-hydroxymugineic acid, 3-*epi*-hydroxymugineic acid, avenic acid, 2’-hydroxyavenic acid	Amino acid metabolism (methionine)	Mobilization, complexation and re-uptake of complexed Fe, (Zn, Cu) in strategy II species	TOM1	All grass species	Nozoye et al. (2011); Römheld and Marschner (1990); Ueno et al. (2007)
**Organic acid anions:** Citrate, malate, oxalate, shikimate, malonate, acetate, citramalate, salicylate	Central carbon metabolism, (and others)	Mobilization of P via ligand exchange or ligand promoted mineral dissolution	MATE?, ABC?, ALMT1^1^	All species but particularly cluster root forming species	Jones et al. (2003); Khorassani et al. (2011); Oburger et al. (2013); Playsted et al. (2006)
**Phenolic methyl ester:** Methyl 3-(4-hydroxyphenyl) propionate (MHPP)	unknown	Biological nitrification inhibition, alteration of root system architecture affecting plant nutrient uptake	MATE? ^1^	*Sorghum bicolor*	Nardi et al. (2013); Subbarao et al. (2015)
**Proteins:** Acid phosphatases	Amino acid metabolism	Hydrolysis of organic phosphate esters	Exocytosis	*Caustis blakei, Lupinus albus*	Playsted et al. (2006) Wasaki et al. (2009)
**(d) Drought stress & soil structure**					
**Mucilage:** Polysaccharides, lipids, proteins	Amino acid metabolism, fatty acid metabolism, central C metabolism	Slowing down of breakup of the liquid phase due to high viscosity of mucilage during soil drying	Exocytosis	Presumably all species	Carminati et al. (2017)
**Mucilage—Polysaccharide:Xyloglucan:** Polysaccharide Xyloglucan	Amino acid metabolism, fatty acid metabolism, central C metabolism	Inducing soil particle aggregation	Exocytosis	*Triticum aestivum, Zea mays, Hordeum vulgare, Pisum sativum, Solanum lycopersicum, Brassica napus, Arabidopsis thaliana*	Galloway et al. (2018)
**Phosphate ester:** Glycerol-3-phosphate	Central C metabolism	Selection of drought tolerant microbiome (monoderm bacteria) improving crop drought tolerance	GP3-Permease	*Sorghum bicolor*	Xu et al. (2018)
**(e) Microbial community composition in the rhizosphere**					
**Aromatic acid:** Salicylate	Amino acid metabolism- shikimate pathway isochorismate Synthase/ Phenyl-alanine-ammonia-lyase (PAL) pathway	Modulation of root-associated microbial communities	Unknown	*Arabidopsis thaliana*	Berendsen et al. (2018); Kniskern et al. (2007); Lebeis et al. (2015)
**Benzoxazinoids:** DIMBOA-Glc, DIMBOA, HDMBOA-Glc	Amino acid metabolism – tryptophan biosynthesis – indole metabolism	Selective impact on rhizobiome: depletion of *Flavobacteriaceae* & *Comamonadaceae* and enrichment of various potential pathogenic funigi	Unknown^2^	*Zea mays*	Cadot et al. (2021)
**Coumarins:** Scopoletin, fraxetin, sideretin	Phenylpropanoid biosynthesis	Limiting growth of *Pseudomonas* in a synthetic rhizobiome community by generating ROS affecting microbial proliferation	ABCG	*Arabidopsis thaliana*	Voges et al. (2019)
**Glucosinolates:**	Amino acid biosynthesis	*Alphaproteo bacteria*, *Rhizobiaceae*, and fungal communities were altered in both structure and composition	PEN3 (ABCG) interacting with PEN2 (Myrosinase)	*Arabidopsis thaliana*	Bressan et al. (2009)
**Glycoalkaloid saponin:** Tomatine	Phenylpropanoid biosynthesis – cholesterol biosynthesis (not fully resolved)	Enrichment of *Sphingomonadaceae* in tomato rhizosphere	Unknown	*Solanum lycopersicum*	Nakayasu et al. (2021)
**Oxylipin:** Jasmonate	Lipid metabolism	Modulation of root-associated microbial communities	Unknown	*Arabidopsis thaliana*	Berendsen et al. (2018); Carvalhais et al. (2015); Doornbos et al. (2011)
**Ureides:** Allantoin	Amino acid biosynthesis (glutamine), purine catabolism	Increase in *Clostridium* and *Sporosarcina* and decrease in *Gracilibacter, Opitutus, Pelotomaculum, Phenylobacterium* and *Oxobacter* in rice rhizosphere under both high and low P	Ureide permease (UPS)	*Oryza sativa*	Lescano et al. (2020); Matsushima et al. (2021)
**Vitamin:** Pantothenate	Amino acid biosynthesis (valine, β-alanine)		Unknown		
**Non-proteinogenic amino acids:** 2-Aminobutyrate (GABA)	Amino acid biosynthesis (glutamate, proline), polyamide pathway		ALMT1		
**Hexosamines:** N-Acetylglucosamin (GlcNac)	Amino acid and central carbon (glucose) metabolism		Unknown		
**(f) Plant-plant interaction**					
**Ureide:** Allantoin	Purine catabolism	Stimulation of germination and growth of barnyard grass (*Echinochloa crus-galli*)	Ureide permease (UPS)	*Oryza sativa*	Sun et al. (2012)
**Diterpene:** Momilactone A & B	MEP pathway;	Allelopathic effect on weed growth, e.g. herbicide resistant barnyard grass	Unknown	(allelopathic) *Oryza sativa*	Kato-Noguchi et al. (2008); Kato-Noguchi and Peters (2013) Yang et al. (2017)
**Flavonoid:** Tricin	Shikimate and MEP pathway				
**Benzoxazinoids (BX):** DIBOA, DIMBOA **BX degradation products:** APO, AMPO	Amino acid metabolism tryptophan biosynthesis – indole metabolism	Root growth inhibition in *Avena fatua, Lolium rigidum, Arabidopsis thaliana, Lactuca sativa* through inhibition of histone deacetylation	Unknown^2^	Cereal crop species	Macías et al. (2006); Venturelli et al. (2015)
**Benzoquinone:** Sorgoleone	Fatty acid metabolism & amino acid metabolism (methionine)	Allelopathic effect on weed seedling growth (weed seedlings tested: *Abutilon theophrasti, Datura stramonium, Amaranthus retroflexus, Setaria viridis, Digitaria sanguinalis, Echinochloa crus-galli*)	Exocytosis	*Sorghum bicolor*	Einhellig and Souza (1992)
**Carotenoid:** (-)-Loliolide	Carotenoid biosynthesis	Increase in DIMBOA concentration in neighbouring wheat roots; induced expression of momilactone B and tricin in seedling roots of allelopathic rice	Unkown	*Triticum aestivum, Eleusine indica, Digitaria sanguinalis, Abutilon theophrasti, Bidens frondosa, Lolium perenne, Avena fatua, Alopecurus japonicus, Aegopilus tauschii, Erinocholoa crus-galli*	Kong et al. (2018); Li et al. (2020b)
**Oxylipins:** Jasmonate	Lipid metabolism				
**Carotenoid-derivatives:** Strigol, solanacol, sorgomol, orobanchol, sorgolactone, 4-deoxyorobanchol, 5-deoxystrigol	Methylerythritol phosphate (MEP) pathway, carotenoid pathway	Germination factor of root parasitic *Striga, Orobanche* and *Phelipanche spp*. with strong negative effect on yield	ABC	*Sorghum bicolor, Oryza sativa, Pisum sativum, Solanum lycopersicum*	Floková et al. (2020)

^1^ Suggested for this compound class by Sasse et al. (2018) and references therein

^2^ Inactive BX-glucosides are stored in plant cells within vacuoles, preventing autotoxicity. A proposed model suggests that BXs are released into the apoplastic space as BX-glycosides and later transformed into bioactive aglycones by extracellular glucosidases (Ahmad et al. 2011). Therefore, BX secretion is presumably (at least partially) driven by vesicle fusion and exocytosis. A recent proteome profiling indicated the presence of an extracellular DIMBOA-β-glucosidase in wheat root tip mucilage (Staudinger et al. 2022)

Nevertheless, considering the wide range of soil/rhizosphere processes driven by root exudates, we should continue in our efforts to reveal the quality and quantity of exudates released along the root axis over time as this knowledge is a prerequisite to deciphering mechanisms of individual exudate compounds and their feedback loops. Linking exudate identity with a specific rhizosphere mechanism will ultimately allow us to improve plant-breeding efforts to harness the benefits of exudate-driven belowground processes. However, studies investigating exudate quality and quantity under natural (soil) growth conditions are still rather limited. Due to the complexity of soil structure, root system architecture and the multitude of processes that are immediately activated once the plant root releases C compounds into the soil, sampling of root exudates unaltered by these processes from soil-grown plants is highly challenging (Oburger and Jones 2018). However, if we want to link root exudation to rhizosphere processes, it is imperative to collect exudates from soil grown plants in situ as plant metabolism (and therefore root exudation) will be significantly affected by the growth medium and its associated microbiome.

## Carbon turnover and C sequestration

Soil C content and related soil C dynamics are an integral factor of soil health affecting plant growth and performance. Together with leaf litter, root litter (i.e. dead roots and associated hyphae) poses a major C input into the soil, particularly for subsurface horizons. In addition, living roots and mycorrhizal hyphae and associated microbiota actively distribute C throughout the soil via the release of root exudates, mucilage, sloughed-off root cells and cell wall debris, depositing C into soil pores and onto mineral surfaces contributing to soil organic carbon (SOC) stabilization (Frey 2019). Consequently, roots play a central role in the dynamics of SOC pools and fluxes as well as in soil structure formation. While the importance of the input and stabilization of SOC by roots is increasingly recognized, paradoxically, SOC destabilization by plant roots has also been observed to play a crucial role in soil C dynamics (Dijkstra et al. 2021). Roots were found to not only form but also destroy aggregates, rendering previously protected C available to microbial decomposition (He et al. 2020; Six et al. 2000). Furthermore, roots and related rhizodeposition are known to change soil organic matter decomposition dynamics when compared to rootless soil under the same environmental conditions, which is generally referred to as the rhizosphere priming effect (RPE) (Kuzyakov 2002). RPE can lead to both accelerated but also reduced SOC destabilization, with reported changes in decomposition rates ranging from 50% reduction to 380% increase (Cheng et al. 2014). Possible mechanisms driving the RPE comprise (i) increased microbial growth and activity due to rhizodeposition resulting in an increase of co-metabolic SOM decomposition (microbial activation hypothesis), (ii) reduced mineral N due to plant uptake promoting N mining from SOM thereby increasing SOM decomposition (microbial N-mining hypothesis) and (iii) the aforementioned physical destruction of macroaggregates by roots exposing previously protected SOM to microbial decomposition (aggregate destruction hypothesis) (Lu et al. 2019; Vetterlein et al. 2020). In a recent review Dijkstra et al. (2021) proposed a framework termed” Rhizo-Engine” to reconcile the paradox of both SOC stabilization and destabilization co-occurring in the rhizosphere. The authors identified two key components driving SOC stabilization and destabilization; microbial turnover and the physicochemical soil matrix. Microbial turnover can be fueled by plant litter and rhizodeposition, as well as from unprotected but also protected SOC pools leading to SOC mineralization but at the same time producing microbial necromass. Reactions of the physicochemical soil matrix with various SOC pools are responsible for protection/stabilization as well as for deprotection/destabilization of SOC, with the latter feeding again into microbial turnover. Root activity can further accelerate or decelerate SOC stabilization/destabilization by (i) physical and chemical liberation of C by rhizodeposition, (ii) formation and destruction of aggregates as well as by (iii) water and nutrient uptake. While this framework helps us to grasp the interlinked complexity of these co-occurring processes in the rhizosphere, the net effect of C sequestration and nutrient turnover will ultimately depend on plant economic traits, symbiotic relationships between plants and microbes as well as environmental factors such as soil properties and climatic conditions (Bastida et al. 2019; Dijkstra et al. 2021; Henneron et al. 2020). Currently we have only started to understand global dynamics and resulting effects of site-specific rhizosphere dynamics and their impact on C and nutrient cycling.

## Plant nutrition

Sufficient nutrient availability is essential for plant health and yield. However, our focus must lie not only on crop quantity but also on crop quality. Mineral and vitamin malnutrition – the so-called “hidden hunger” is considered one of the greatest challenges currently faced by human kind. The World Health Organization estimates that two billion people suffer from micronutrient malnutrition, like iron (Fe) and zinc (Zn), causing 7.3% of disease burden (Thompson and Amoroso 2014). Plant breeding-based biofortification, i.e. the delivery of micronutrients via micronutrient-rich crops, is considered the most cost-effective and sustainable approach to alleviate this hidden hunger (Welch and Graham 2004; White and Broadley 2009). Traditional interventions like mineral supplementation, industrial fortification, crop fertilization, etc., require infrastructure and access to markets and therefore often fail to reach the most vulnerable people in remote areas.

Growing (micro)nutrient-efficient crops is of particular importance in arid and semi-arid environments that are dominated by high pH and/or saline soils that are typically characterized by low and unbalanced phytoavailability of nutrients. At high pH nutrients are strongly fixed either by precipitation (Fe, P) or (particularly relevant for cationic micronutrients) by sorption to negatively charged mineral surfaces (White and Broadley 2009). Furthermore, nutrient imbalances also occur in high acidic soils where plants are challenged with Ca, P and Mo deficiency as well as with Fe, Al, and Mn toxicity (Adams 1981). While translocation of nutrients within the plant tissue, especially to the edible parts during ripening, is an important aspect of (micro)nutrient efficiency in crops, the first and most important barrier to nutrient absorption resides at the root-soil interface (Bishopp and Lynch 2015). Differences in root architecture and geometry have been found to play an important role in nutrient acquisition. For example, shallow basal root growth enhances topsoil foraging for phosphorus (P) because in most soils P is concentrated in the topsoil (Lynch and Brown 2001). In addition, root hairs are implicated to increase the absorption surface of the root and therefore the volume of soil that can be scavenged for nutrients (Gahoonia and Nielsen 2004). Miguel et al. (2015) demonstrated that a shallow basal root growth angle together with high root hair length and density had a synergistic effect on P acquisition efficiency in common bean (*Phaeseus vulgaris*) and resulted in increased growth and P uptake compared to common bean lines either lacking in or expressing only one of investigated root phenotypes. However, a shallow root architecture was found to be less efficient in capturing N and it can also be a disadvantage under drought stress due to top soil drying. Dimorphic root phenotypes combining deep rooting with shallow rooting of the top soil are considered as more favorable particularly in climates facing drought periods (Lynch 2019).

Plant belowground traits relevant for nutrient acquisition are not limited to different root architectural and morphological phenotypes, but also include the ability of plants to shape rhizosphere properties to their benefit. Roots can enhance nutrient availability either directly via the exudation of nutrient solubilizing compounds (e.g. protons, carboxylates, enzymes, phytosiderophores, coumarins)(Dakora and Phillips 2002) or indirectly by sustaining a microbial community that efficiently solubilizes mineral nutrients and incorporates them into their biomass (Sasse et al. 2018). Furthermore, though temporarily unavailable, nutrients stored in the microbial biomass are generally considered plant available due to rapid microbial turnover in the rhizosphere (Raymond et al. 2021). While changes in root morphology, transporter expression and activity alter the plants’ nutrient uptake capacity, the modification of nutrient solubility via rhizosphere processes ultimately determines the pool size of nutrients available for uptake. A crop with a high nutrient absorption capacity will still grow poorly if soil nutrient availability is insufficient. Consequently, it will be of crucial importance in the future to improve our understanding of root traits and related rhizosphere processes and support plant breeders in selecting crops that grow well (in terms of quantity and food quality) under nutrient poor conditions.

The nutrient solubilizing capacity of specific exudate compounds like organic acid anions, coumarins, and phytosiderophores (grass species only) has been repeatedly demonstrated (e.g. Baune et al. 2020; Oburger et al. 2011; Schenkeveld et al. 2014; Schmid et al. 2014; Walter et al. 2016, see also Table 1) and reviewed (Adeleke et al. 2017; Dakora and Phillips 2002; Jones and Darrah 1994). While mechanistic studies provide important insights on the concentration-dependent nutrient mobilizing potential of exudates, there is still a lack of data on whether or not exudation rates, particularly from soil grown plants, are high enough to induce sufficient nutrient mobilization. Probably the best reported example of successful, exudate-driven nutrient acquisition includes P mobilization by all cluster and dauciform root forming species from the families *Proteaceae, Restionaceae, Cyperaceae* and *Fabaceae* (Lambers et al. 2008, 2006). Cluster roots are only a few days physiologically active, during their development (juvenile stage), they accumulate high concentrations of organic acids in their tissue which they then release as organic acid anions together with protons and acid phosphatases at maturity in an exudative burst into the soil allowing for highly efficient P solubilization even in P impoverished soils (Playsted et al. 2006). Interestingly, in contrast to several reports on the high P acquisition efficiency of cluster roots (Lambers et al. 2008 and references therin), Gusewell and Schroth (2017) did not find differences in nutrient acquisition or evidence for nutritional niche differentiation of European* Carex* species with and without cluster roots grown in a semi-hydroponic system. Hydroponic experiments with *Brassica* also observed an increase in organic acid exudation upon P starvation (Akhtar et al. 2008; Aziz et al. 2011; Shahbaz et al. 2006). Similarly, screening phytosiderophore exudation of different bread (*Triticum aestivum*) and durum (*T. turgidum L. conv. durum*) genotypes in hydroponics under Zn deficiency suggested higher phytosiderophore exudation rates for Zn efficient genotypes (Cakmak et al. 1994; Rengel 1999; Rengel and Römheld 2000). While reported evidence based on hydroponic studies is promising, soil-based studies are needed to verify these results under natural growth conditions.

Changes in rhizosphere pH and redox potential are also known to affect nutrient solubility at the root-soil interface (Hinsinger et al. 2003). The underlying physiological mechanisms driving rhizosphere acidification/alkalinization are complex and can be caused by multiple, potentially co-occurring factors. Proton release pathways have been well described with H^+^-ATPase being the major proton transport plasma membrane protein (Yan et al. 2002; Zhu et al. 2009). While the release of OH^−^ or HCO_3_^−^ is often proposed as the dominant mechanisms leading to rhizosphere alkalinization (Hinsinger et al. 2003 and references therin), to the best of our knowledge, a transport mechanism for OH^−^ or HCO_3_^−^ has not yet been identified. Irrespective of the precise mechanisms, one major driver of changes in rhizosphere pH has been found to be the cation–anion uptake balance. Especially the ionic form of mineral nitrogen (NO_3_^−^, NH_4_^+^) uptake was found to have a major impact on rhizosphere pH (Hinsinger et al. 2003; Kirkby and Mengel 1967; Kosegarten et al. 1997; Ruan et al. 2000). The effect of N forms on cellular pH homeostasis and the current understanding of how changes in rhizosphere pH are brought about, have been extensively reviewed elsewhere (Britto and Kronzucker 2005; Feng et al. 2020). Briefly, expression and activity of H^+^ pumping complexes, such as plasma membrane H^+^-ATPase, are upregulated upon uptake of NH_4_^+^ leading to rhizosphere acidification while NO_3_^−^ is taken up by plant roots via a 2H^+^/ NO_3_^−^ symporter. This was further confirmed when *Arabidopsis* plants with a point-mutation in the gene encoding the major nitrate transporter NRT1.1 showed no rhizosphere akalinization when grown on NO_3_^−^ rich medium (Fang et al. 2016). Marschner and Römheld (1983) nicely demonstrated that the extent of pH changes very much depended on the level of either NO_3_^−^ and NH_4_^+^ applied but also differed between species and was influenced by soil pH buffer capacity. Species specific differences were suggested to be linked to the different responses of H^+^-ATPases activity upon NH_4_^+^ (cation) uptake (Schubert and Yan 1997). Depending on the driver, Römheld et al. (1984) also observed either acidification of the entire root system due to the preferential uptake of NH_4_^+^ and K^+^ by hydroponically grown sunflower seedlings or intensive proton release at the root tips only upon Fe deficiency. Santi and Schmidt (2009) did not only decipher the underlying molecular mechanisms of Fe-deficiency induced proton release in *Arabidopsis*, they also reported differences in acidification capacity among *Arabidopsis* accessions indicating a genotypic diversity in Fe acquisition efficiency that is linked to the extent of rhizosphere acidification in all non-grass species (strategy I). Nutrient deficiency induced rhizosphere acidification (Nussaume et al. 2011; Xu et al. 2012; Yan et al. 2002) but also alkalinization (Kuppe et al. 2022) has also been reported upon P starvation and considerable acidification is typically found in the rhizosphere of N_2_ fixing legumes (Marschner and Römheld 1983). Intercropping with legumes therefore might not only improve N nutrition in the co-crop but can also have a positive effect on P and micronutrient uptake (Gunes et al. 2007). In addition, growth of young root tissue and root hairs also seem to be generally accompanied by the release of protons (Bibikova et al. 1998; Hager 2003) and can consequently affect rhizosphere pH and nutrient solubility. Even though much progress has been made on revealing the mechanisms of rhizosphere pH changes, there is only a limited number of studies looking at genotypic differences in rhizosphere acidification/alkalinization and whether or not these differences translate to higher nutrient acquisition efficiency. Screening 10 chickpea (*Cicer arietum*) genotypes in a calcareous soil as well as in nutrient solution culture, Gahoonia et al. (2007) observed a higher absorption of Fe, Zn, Mn, K and P by genotypes inducing stronger acidification and possessing longer and denser root hairs. Interestingly, screening 10 lentil (*Lens culinaris*) lines grown on the same calcareous soil, Gahoonia et al. (2006) could only link prolific root hair formation with enhanced nutrient uptake but observed no differences in rhizosphere acidification.

Taken together, all these findings suggest that both, root exudation and the modulation of rhizosphere pH might be promising plant traits in crop breeding programs. However, more in-depth work relating genotypic differences in root exudation, as well as in rhizosphere acidification/alkalinization under varying environmental (soil) conditions to nutrient uptake is needed to successfully capitalize on these plant traits in the future.

To date, several approaches to improve plant nutrition and growth performance exist that are already capitalizing on beneficial plant–microbe interactions. Most plants on land (about 90%) are forming a symbiosis with mycorrhizal fungi, trading photosynthates for fungal-acquired nutrients (Averill et al. 2019; Tedersoo et al. 2020). Based on their structure and function, four major mycorrhizal types have been described, namely arbuscular mycorrhiza (AM), ectomycorrhiza (EM), orchid mycorrhiza and ericoid mycorrhiza. About 75% of plants are estimated to form AM associations, 2% of plants are colonized by EM, about 9% of plants form orchid mycorrhiza and ca. 1% of plants form ericoid mycorrhiza (Brundrett 2002). It is rather the rule than the exception that an individual plant is infected by multiple mycorrhizal fungi and most mycorrhizal fungi are not host-specific. Some plant species like poplars and eucalypts, also form dual mycorrhizal associations with AM and EM fungi (van der Heijden et al. 2015). The same authors summarized that mycorrhiza can acquire between 70%-100% of plant phosphorus (P) uptake (irrespective of mycorrhiza type) and contribute up to 20% (AM) and 80% (EM, Ericoid) respectively to plant nitrogen (N) acquisition. While EM were reported to dominate particularly temperate and boreal forest ecosystems, AM are most relevant from an agronomic point of view, as many crops form associations with AM (Read 1991). However, there is an ongoing debate whether or not farmers should actively modify their management in order to enhance the abundance and diversity of AM (Rillig et al. 2019; Ryan and Graham 2018). Likewise, there is continuous critical discussion on whether the benefit of applying industrial fungal bioinoculants outweighs the risk of additional financial expenses, as well as potential negative effects on plant and soil diversity and ecosystem functioning (Hart et al. 2018). While positive mycorrhizal growth responses have been reported many times from controlled laboratory and greenhouse experiments, results from field studies are less clear (but also far less abundant). This arises from the complex interactions of numerous, partly uncontrollable factors that can influence plant growth in the field that complicate identifying reliable mechanistic drivers of growth responses (Ryan and Graham 2018). However, a meta-data analysis showed that plant response to mycorrhizal colonization is most positive when plants are P limited rather than N limited (Hoeksema et al. 2010). The authors further revealed that woody plants, non-N fixing forbs and C4 grasses responded more positively to mycorrhizal inoculation than plants with N-fixing bacterial symbionts and C3 grasses. In addition, a negative relationship between AMF benefits and root hair length has been reported (Schweiger et al. 1995). Depending on relative supply of P and N and probably also other nutrients, as well as levels of water availability and light, the relationship between plant and mycorrhizal fungi was found to range from mutualism to commensalism to parasitism, which is also referred to as the so-called trade-balance model (Johnson et al. 1997; Johnson and Graham 2013). Nitrogen nutrition generally seems to play a crucial role in determining the agronomic success of mycorrhizal colonization, with low N availability often resulting in N competition between plants and AM leading to a negative growth response (e.g. Püschel et al. 2016). Research of the past decades indicates that achieving a positive yield response by increasing AM colonization or adding industrial inoculants very much depends on plant species and environmental conditions, and our current knowledge is insufficient to reliably predict successful application/management scenarios (Ryan and Graham 2018). Nevertheless, as highlighted by Rillig et al. (2019), next to their direct influence on plant nutrition/yield, mycorrhiza also provide several other, highly relevant ecosystem services, including soil organic matter decomposition and stabilization, reduction of N leaching losses, denitrification and reduced N_2_ losses, regulation of plant diversity, as well as increasing soil aggregation and plant seedling survival. Another recent study suggests that inoculation of fungi could generally result in short-term increased plant productivity. This, however, comes at a potential cost of reducing biodiversity by anthropogenically increasing the abundance of mutualistic fungi that provide less of these ecosystem services noted above (Martignoni et al. 2020).

Next to mycorrhiza, a range of other, free-living microorganisms (e.g. several species of the genera *Pseudomonas*, *Aspergillus* or *Penicillium*) have been identified to efficiently solubilize phosphate from which plants can potentially benefit (Richardson 2001). The use of free-living phosphate solubilizing microorganisms (PSM) as biofertilizers has been intensively investigated in the past decades ranging from *in vitro* experiments, to controlled laboratory/greenhouse studies, to field trials. Microbial P solubilization mechanisms include dissolution of P minerals via acidification, ligand exchange as well as ligand promoted mineral dissolution by released carboxylates, as well as the release of extracellular phosphatases that transform organic P to inorganic P which can then be taken up by neighbouring plants (Jones and Oburger 2011; Rodríguez and Fraga 1999). A recent study demonstrated that PSM can feed on plant-derived pectin which is the major polymer of root mucilage and primary cell walls (Mise et al. 2020). Interestingly, microbial genes associated with pectic lyase activity were significantly increased in P deficient tropical soils (Yao et al. 2018), suggesting that PSM can establish in the rhizosphere and that plant root-derived pectin contributes to patterns in rhizosphere microbial community assembly. Similar to mycorrhiza, positive plant growth responses to inoculation with PSM were mostly reported for controlled laboratory conditions (e.g. Pande et al. 2017; Wakelin et al. 2007), while field trials (but also laboratory studies) more frequently failed to demonstrate an increase in plant growth or yield (Karamanos et al. 2010; Meyer et al. 2017; Raymond et al. 2019). In addition to important factors determining the colonization and persistence of PSM in soils (pH, P, N, C availability, inoculum quality and placement strategy), Raymond et al. (2021) summarized that PSM generally do not have the capacity to solubilize sufficient P beyond meeting their own need to improve the crops P supply on a short term scale. The authors suggested that future mechanistic studies on P mobilization by PSM should focus on PSM as a component of the whole soil community addressing the longer-term role of P storage and cycling by the soil microbiome.

Biological nitrogen fixation (BNF) is another important rhizosphere process that we can harness to improve plant nutrition. BNF is carried out by bacteria capable of fixing atmospheric nitrogen (N_2_) and transforming it into ammonia (NH_3_) via the nitrogenase enzymes. In soil, these specialized bacteria either occur as free-living bacteria (e.g. *Azotobacter*), form associative relationships with host plants (e.g. *Azospirillum, Kosakonia*), or they can establish symbiotic associations with legumes and other plant species (e.g. *Rhizobium* harboured in nodules). In the latter, the plants provide photosynthetic C while the bacteroids deliver nitrogen fixed from the atmosphere to the host plants. It has been estimated that the global contribution of symbiotically fixed N_2_ is likely to be in the order of 20–22 million tons N per year (Herridge et al. 2008). Since the quantity of symbiotically fixed N is directly related to plant growth performance, factors affecting host plant biomass production such as water and nutrient availability or disease incidence and pests are crucial determinants of the amounts of N_2_ fixed. Furthermore, agricultural practises affecting effective rhizobia in soil or soil nitrate concentrations (excessive tillage, application of N fertilizer) were found to be critical (Peoples et al. 2009). Host infection by an appropriate rhizobial strain is usually most effective when the host plant was part of a recent crop rotation otherwise desired strains might be absent and inoculation is needed to ensure satisfactory nodulation. Furthermore, the timing of inoculation has been reported to affect the contribution of BNF to crop growth. Re-inoculation of soybeans with *Bradyrhizobium* strains at several plant growth stages significantly increased the amount of N provided by inoculated diazotrophs and also was found beneficial for grain yield and N content in grains (Hungria et al. 2006). Comparing yield responses upon inoculation to local farming practices in 377 field trials with different legume crops from more than 20 countries, Peoples et al. (2009) found that on average 57 ± 21% (mean ± SD) trials had a significant positive yield response upon inoculation. These findings highlight the potential but also the challenges of symbiotic BNF in sustainable agriculture. The same authors also pointed out that poor inoculum quality, in addition to lack of knowledge/training and financial means particularly in Africa and Asia is often responsible for no yield responses. As economic restrictions remain an insurmountable problem particularly in developing countries, plant breeding and research efforts should also focus on promiscuously-nodulating legume lines that require no inoculation by farmers. Additionally, other soil management and agronomical practices need to be further explored to maximize N inputs by symbiotic BNF including use of legume genotypes best adapted to prevailing soil and environmental conditions, including optimized regional planting time, incorporation of legume residues, intercropping, cereal-legume crop rotations, use of short duration legume green manure or legume catch crops (Peoples et al. 2009). Such a multi-faceted strategy was followed in the N2AFRICA project, a science-based endeavour aiming to enhance productivity of smallholder farmers in Africa by growing legume crops (https://www.n2africa.org/home; Giller et al. 2013). The inclusion of additional forms of capacity building such as education, women’s empowerment, and improved access to local markets represents a promising template for future strategies to expand legume-rhizobia symbioses in sustainable agriculture.

Besides nodule-forming rhizobia, inoculation formulae containing associatively-living N_2_ fixing strains have also been investigated in field trials for decades. Okon and Labandera-Gonzalez (1994) reviewed the application of *Azospirillum* inoculates, a rhizobacterium known for its associative N_2_ fixation, in agriculture after 20 years of worldwide field application. Back then, the authors reported success rates of 60–70% with statistically significant yield increases by 5–30%. Similar to *Rhizobium* inoculates, the quality (optimal number of viable cells) of *Azospirillum* inoculum played a crucial role in achieving a positive yield effect. Research in the past decades however revealed that improved plant growth after inoculation with *Azospirillum* is most likely more related to its capacity to produce phytohormones, like indole-3-acetic acid, than to its N_2_ fixation activity (Fukami et al. 2018). The contribution of associative N_2_ fixation to N nutrition of crops has been observed to be most pronounced for C4 plants in soils of subtropical and tropical climates where enough C can be provided by the host plant to support the energy-demanding process of N fixation by the inoculated diazotrophs (Dobbelaere et al. 2001). In recent years, potential strategies to harness N fixed by free-living bacteria in the rhizosphere have been put forward and discussed multiple times (e.g. Smercina et al. 2021; Bennett et al. 2020; Bloch et al. 2020). A potentially promising avenue represents the editing of the genome of bacterial strains via synthetic biology to increase their N fixation capacity under field condition and to render a commercial application as bio-fertilizers a successful endeavour. Such an approach was reported for a free-living strain of *Klebsiella variicola*, where the authors replaced the *nifL* gene, which usually represses N fixation under conditions of sufficient N availability, with a constitutive promoter to obtain a strain that could still fix N under field conditions (Wen et al. 2021). In a number of trials where the strain was applied in addition to inorganic fertilizers in corn fields, increased yields of around 3% and lower within-field yield variance were observed as compared to fields that were only subjected to inorganic fertilization. Although such an approach could support a more sustainable intensification of agricultural systems, it remains unclear if the increase in productivity was due to an increased provision of N to the maize plant by the genetically modified strain or due to other plant growth promoting factors such as detailed before. It is also questionable if a moderate increase in yield, and thus a relatively high cost-to-benefit ratio, will be sufficient to stimulate the application of rhizosphere-associated microorganisms in agricultural systems (Shah et al. 2021). Moreover, the long-term impact of genetically modified strains on microbial diversity and related ecosystem functions still remains unknown.

## Plant health

Next to optimal water and nutrient use efficiency, overall plant immunity, defined as the inherent or induced capacity to resist or tolerate pathogens and herbivores, is an important trait affecting overall plant growth and yield. How much resources are allocated to plant growth or to plant defense strategies is precisely regulated by the complex and interconnected crosstalk among phytohormones. Phytohormones are endogenously produced small organic molecules regulating gene expression via signal transduction pathways in response to changes in environmental conditions. Besides the classical groups of phyothormones (auxins, cytokinins, gibberellic acid, ABA and ethylene) (Egamberdieva et al. 2017 and references therein), other compounds have more recently been identified as plant hormones (salicylic acid, jasmonates, brassinosteroids, strigolactones and small peptides (Al-Babili and Bouwmeester 2015; Berens et al. 2017; Kaufmann and Sauter 2019)). On the one side, most phytohormones are involved in plant immunity and regulate rhizosphere microbiome assembly; on the other side, soil microbes themselves can produce certain phytohormones and trigger changes in plant hormonal homeostasis (Eichmann et al. 2021). Reductionist approaches using a small number of microbes under controlled environmental conditions have helped us to identify causal relationships in plant–microbe interactions. Besides constitutively synthesised physical and chemical barriers (cell wall polymers such as suberin and lignin, or antimicrobial phytoanticipins) (Singh et al. 2021; VanEtten et al. 1994), plants can detect microorganisms and trigger complex signaling cascades leading to induced immune responses that confer a more tolerant phenotype. These immune responses include enhanced local production of reactive oxygen species at the infection site (Survila et al. 2016) and improved ROS detoxification in neighboring tissues (Souza et al. 2017)(but also under abiotic stress conditions), increased abundance of pathogen-related proteins, callose accumulation (Millet et al. 2010) or the enhanced synthesis of specialized antimicrobial metabolites (Duan et al. 2014). Low molecular weight (LMW) antimicrobial metabolites that are induced upon pathogen infection but are otherwise not present in healthy plants are generally referred to as “phytoalexins”, while “phytoanticipins” are defensive compounds that are present in plants before being challenged by pathogenic microorganisms and are typically upregulated under pathogen attack (VanEtten et al. 1994). Examples of exuded metabolites that have been identified to act as phytoalexins/phytoanticipins in the rhizosphere are summarized in Table 1.

The activation of these responses is sparked by conserved molecular patterns such as bacterial flagellin, fungal chitin or damage-associated molecular patterns, which are perceived by plasma membrane-associated plant receptors that trigger signaling cascades resulting in basal defense mechanisms (Antolin-Llovera et al. 2014; Souza et al. 2017). In the rhizosphere, beneficial microbes can induce plant physiological changes that result in enhanced growth or stress resistance. Plant-growth promoting rhizobacteria (PGPR) are typically identified by their ability to produce and release phytohormones or induce alterations in plant hormone homeostasis. Inoculation with phytohormone-releasing PGPR can result in increased root growth under nutritional or other environmental stresses (drought, salt, pollution, etc.), leading to improved biomass production as larger root systems allow increased resource acquisition (Fukami et al. 2018; Hayat et al. 2010; Nadeem et al. 2013). Furthermore, specific species of the root-associated genera *Pseudomonas*, *Bacillus* and *Trichoderma* have been shown to enhance plant immunity not only locally at the site of infection, but can also stimulate the defensive capacity in distal plant organs (Pieterse et al. 2014). This phenotype is referred to as induced resistance phenotype and is often mechanistically linked to immune responses regulated by salicylic acid or ethylene and jasmonic acid (De Kesel et al. 2021). For example, root colonization by *Pseudomonas simiae* stimulated the production of glucosinolates in *A. thaliana* leaves via ethylene and jasmonic acid signaling cascades which enhanced overall herbivore resistance (Pangesti et al. 2016).

Bacteria and fungi that are capable of releasing metabolites which trigger plant defence mechanisms against pathogens, insect herbivory, and abiotic stressors are of great interest in crop management. For example, by applying an improved identification and inoculation approach, Mueller et al. (2021) reported a successful selection for rhizosphere microbiomes that confer salt tolerance to the model grass *Brachypodium distachyon* in greenhouse experiments. Treated plants grown under sodium or aluminum salt stress showed an increase of 55–205% in seed production. Although the identification and testing of individual microorganisms or microbial consortia is laborious, its benefits for sustainable agriculture remain a promising avenue to tailor genotype- or environment-specific plant-microbiota interactions leading to beneficial rhizosphere processes. Engineering of such root-associated microbiomes with plant-beneficial traits like phytohormone production could significantly simplify the selection of suitable microorganisms. Currently, multiple approaches to design and apply plant-associated microbiomes are discussed and the outcomes could indeed prove valuable towards more sustainable agriculture (Ke et al. 2021). Especially *in situ* microbiome engineering seems to be a promising approach to add, modify, or delete genes of interest within microorganisms of a natural community. Such a “community editing” tool was recently developed which combines a modified CRISPR-Cas system to manipulate the genetic potential of bacterial species with targeted sequencing to track the fate of these edited cells (Rubin et al. 2021). Although this approach has yet to be tested and applied to plant-associated microbiomes, it could potentially be used to edit selected microorganisms with traits of interest within a rhizosphere community, and thus help improve plant health/growth. How such edited microorganisms and their host plants fare under field conditions and if these approaches indeed translate into sustained higher yields needs to be explored in future research. Additionally, the consequences of introducing genetically modified microorganisms into the environment still remain mostly unknown.

Bridging the gap between reductionist approaches and ecological studies is one of the avenues we have to take towards a better understanding of plant-soil-microbe belowground interactions for sustainable agricultural practices. An elaborate experimental design was recently employed to assess the plant growth-defence relationship as affected by the interaction of different microbial communities (Geisen et al. 2022). This study showed that different soil microbial groups (bacteria, fungi, protists) did not alter plant growth and defense when analyzed individually, but that microbial groups and their interactions could alter the relationship between plant growth and defence. In addition, these microbiome-induced effects differed between plant functional groups (grasses or forbs) and age of the respective plant community, indicating that much remains to be uncovered while approaching agriculturally relevant settings (Bender 2022; Wei et al. 2020).

## Belowground plant-plant interaction

The productivity of species-rich plant communities is typically higher than in less diverse, but comparable systems (Prieto et al. 2015; Wuest et al. 2021). This potential overyielding effect of diverse systems is leveraged in agriculture through the use of intercropping or polyculture systems, where different plant species or varieties are grown simultaneously on the same area of land (Brooker et al. 2015). Intercropping and relay intercropping are management practices that have potential for sustainable intensification of agriculture in low-input as well as high-input farming systems (Li et al. 2020a; Wezel et al. 2014). A meta-analysis of a global dataset on grain-producing intercrops showed that higher yield gains were achieved in the vast majority of cases, especially in systems involving maize, where yield gain was four times higher than in polycultures without maize. Overall, yield increases of 16% to 29% were found, while fertilizer inputs were reduced by 19% to 36% in intercropping systems when compared to monocultures of their components under the same management (Li et al. 2020a).

Conceptual frameworks exist that help in gaining a mechanistic understanding of the processes involved in mixture benefits. The mechanisms underlying enhanced productivity of diverse systems, involve interrelated aspects such as trait complementarity with respect to resource use, pathogen susceptibility and modification of soil quality (Tilman et al. 2014). From an ecological perspective, niche differentiation leads to complementarity in the use of abiotic resources thereby increasing the community-level resource pool available for biomass production. However, resource sharing is another aspect of complementarity in plant nutrient acquisition strategies through which root processes of one component increase the availability of nutrients that would otherwise be inaccessible to other components of the system (Brooker et al. 2015; Homulle et al. 2021; Li et al. 2014). Theoretically, less competition among system components also allows for enhanced allocation of resources to biomass production and crop yield, although current crop cultivars might not be optimised for preferential resource allocation to reproductive tissues in polyculture (Chen et al. 2021). Furthermore, reduced pathogen pressure is achieved if pathogens are specialised and their dispersal depends on host density. Evidence for another mechanism of enhanced pathogen resistance in polyculture compared to monocrops was recently found in rice and durum wheat varietal mixtures of a single species (Pelissier et al. 2021). Basal plant immunity was stimulated in varietal mixtures by the presence of healthy neighbours and experimental evidence indicated that this stimulation was achieved by belowground chemical signals. Finally, species of different plant functional groups such as legumes, grasses and forbs have the potential to enhance soil fertility over time (Furey and Tilman 2021). Increased earthworm abundance was observed in legume-cereal intercropping, which was related to enhanced deposition of organic matter in soils (Schmidt et al. 2003). An average 22% yield advantage of intercropping was reported in long-term experiments along a soil fertility and yield production gradient in northwest China (Li et al. 2021). In this study, intercropping systems composed of maize grown with wheat, legumes and/or oilseed rape, both, overyielding and yield stability, increased over time (10–16 years). This productivity effect was partially explained by changes in soil properties. Soil organic matter and total nitrogen were increased in some experimental sites, whereas increased macroaggregate formation in intercrops was observed consistently across experimental sites. Better soil physical properties can have potential benefits on water infiltration, erosion and nutrient cycling (Six et al. 2004). Despite the above-mentioned benefits of intercrops, current industrial farming technologies, as used in monoculture cultivation, are not suited for application in intercropping systems. In monoculture cultivation systems the use of diverse and adequate crop rotations and cover crops can provide benefits in terms of yield increases (Bowles et al. 2020), soil nutrient availability (Hallama et al. 2018), soil physical properties (reviewed by Griffiths et al. 2020) and soil microbial biomass, activity and diversity (Kim et al. 2019). For the large-scale implementation of polyculture farming practices, custom-made technical solutions have yet to be developed.

Experimental evidence suggests that plants interact with heterospecific and conspecific neighbours through a range of aboveground and belowground signals (Bilas et al. 2021). Differences in light conditions (Huber et al. 2021), touch stimulation of aboveground organs or physical root contact (Elhakeem et al. 2018; Fang et al. 2013), volatile chemicals (Huang et al. 2019) and chemical stimuli via root exudates (Semchenko et al. 2014) trigger responses in neighbouring plants on the level of gene expression, root architecture, plant growth and biomass allocation. Plants release compounds derived from primary and specialized metabolism into the surrounding soil (see also Table 1), where they can act as signals perceived by contemporary neighbours or future generations (i.e. plant-soil feedback, Mariotte et al. 2018). Due to their chemical complexity and variability, root exudates are currently regarded as the main drivers of belowground plant-plant interaction with the potential to provide information about neighbour identity, density and physiological state (Wang et al. 2021b). How and to what extent belowground chemical interactions contribute to mixture benefits and overyielding in polycultures is not well understood, especially in soils. Here we summarize the to date best-studied root exudates for which the molecular mechanisms of plant-plant interaction are (partially) known: Prominent examples of belowground cues stimulating germination of neighbouring plants are strigolactones (Floková et al. 2020), which also induce plant-fungal interactions (Kretzschmar et al. 2012) and allantonin. The latter was also reported to enhance the production of ABA, stimulate jasmonic acid signalling pathways (Takagi et al. 2016) and to generally play a role in mediating plant responses to various environmental stresses (Kaur et al. 2021). The most studied plant derived compounds with phytotoxic properties, generally referred to as allelochemicals, are sorgoleone, benzoxazinoids (e.g. DIBOA, DIMBOA) and momilactone A and B. Sorgoleone accumulates in lipid droplets in specialized root hair cells of *Sorghum bicolor* and is known to inhibit germination of small seeded weeds, mainly due to its inhibitory effect on photosynthetic and mitochondrial electron transport. It is also known for the strong inhibition of carotenoid biosynthetic pathways and the inhibition of root H^+^-ATPase which can lead to reduced plant mineral and water uptake (Dayan et al. 2010). Benzoxazinoids are shikimic acid-derived specialized metabolites found in most cereal crop species and some dicot taxa (Frey et al. 2009). Upon release into soils, they are rapidly degraded into the more stable derivatives APO, MBOA and AMPO (Fomsgaard et al. 2004). The phytotoxic activity of benzoxazinoids and their degradation products, was related to chromatin modifications mediated by inhibition of histone deacetylation (Venturelli et al. 2015). However, it has to be noted that benzoxazinoids, as documented for many specialized metabolites, fulfil multiple roles within the plant body and the rhizosphere (for examples, see Table 1). In soils they function as defence compounds and as mobilisers of essential micronutrients. However, benzoxazinoid-iron complexes in soils also can attract insects and a recent study showed that whether benzoxazinoids act as defence chemicals or attractants is context-dependent and strongly influenced by soil chemistry (Hu et al. 2021). The diterpens momilactone A and B are the major allelochemicals found in rice, which are synthesised when allelopathic rice cultivars grow in proximity to heterospecific neighbours or other rice cultivars. The current knowledge on momilactones has recently been reviewed by Serra Serra et al. (2021). The authors conclude that while detailed knowledge on the biosynthetic pathway of momilactones biosynthesis has been obtained, the mechanisms involved in induction, release and phytotoxicity momilactone are not well understood and warrant further investigations. The plant-derived compounds loliolide and jasmonic acid (JA) are found in the rhizospheres of most plants. These ubiquitous chemicals have been shown to elicit defensive responses in neighbours: enhanced momilactone B biosynthesis was observed in rice and the expression of genes involved benzoxazinoid biosynthesis in wheat and rice was significantly upregulated upon exposure to loliolide and JA (Kong et al. 2018).

This handful of examples highlights the diversity and the complexity of compounds and mechanisms involved in plant-plant interaction. Despite our progress in identifying biologically active root exudates and deciphering the related responses in conspecific and/or heterospecific neighbours, we are still a long way from having a comprehensive picture of all relevant agents and involved mechanisms. One single plant species synthesises over 5000 metabolites and an estimated 100000 to 1 million different compounds are to be found in the entire plant kingdom (Alseekh and Fernie 2018). Over 1000 features are commonly detected in root exudate samples, of which approximately 100 compounds can be routinely identified using contemporary metabolomic profiling approaches (van Dam and Bouwmeester 2016). Furthermore, plant-plant interaction studies are not trivial and experiments have to be carefully designed (Bilas et al. 2021). Future research also needs to reveal the influence of rhizosphere properties, including the rhizosphere microbiome, on the transmission and modulation of such chemical cues. Therefore, much remains to be uncovered in terms of (i) identification of new bioactive compounds involved in belowground plant-plant interactions, (ii) describing their dynamics in soil (mobility, dissipation time, effective concentrations) and related to this (iii) the biotic and abiotic functionality of root-released compounds in relation to soil chemistry. These insights will provide a more complete picture allowing for the spatiotemporally optimal management of species and varietal interactions in polycultures under future climate scenarios.

## Conclusion

Rhizosphere processes are governed by plant phenotypic traits including internal water and nutrient use efficiency, systemic and local immune responses as well as root architecture and root resource acquisition efficiency. In turn, associated belowground plant–microbe-soil interactions can significantly affect the phenotypic plasticity of plants. Combining in-depth knowledge of above- and belowground plant traits will therefore allow for more informed decisions regarding sustainable agricultural practices and plant breeding strategies. Next to root architecture and root resource acquisition efficiency, root exudates are considered key drivers of interactions at the plant–microbe-soil interface. Consequently, a major focus currently lies on deciphering exudate diversity and linking individual exudates to processes occurring in the rhizosphere. Especially rhizosphere-associated microorganisms represent a fascinating resource to sustain plant growth and potentially increase the stress resistance and overall productivity of their host plant. The rhizosphere microbial community can either be manipulated by selecting specific genotypes with exudation traits that trigger the establishment of symbiosis and/or favour certain microbial taxa or by applying inoculation solutions either directly to the soil or as plant seed coatings. To date, limitations surrounding inoculation applications on a large scale, including a high cost-to-benefit ratio, country-dependent legal and regulatory affairs, as well as farmer’s scepticism in the face of lacking practical evidence diminish the promising potential of PGPR in agricultural systems. Nevertheless, novel approaches such as genome-editing of single bio-inoculants, whole rhizosphere-associated communities, or microorganisms in conjunction with a specific host plant are under development and could turn the tide towards a more effective implementation of microbe-assisted strategies in sustainable agriculture. At the same time, however, it is imperative to gain a better understanding of potential ecological consequences of introducing new microbiota into environmental systems.

Considering the current unsolved challenges of manipulating the rhizobiome and the involved costs, an alternative and universally applicable approach would be to breed for crops with well-adapted plant traits including root and rhizosphere properties as an extended (belowground) phenotype. While the idea is promising, following through will require a great scientific effort that needs to include mechanistic and applied studies as well as highly differentiated, interdisciplinary approaches that are tailored to different species/genotypes grown in several environmental conditions. While significant progress in understanding rhizosphere processes has been made in the last decades, past research often worked with simplified or artificial systems to break down the great complexity of the plant–microbe-soil environment and allow the identification of underlying mechanisms. Experiments using simplified systems were and still are crucial for stepwise elucidation of belowground interactions, however our advances in both, in-depth knowledge and experimental techniques, now enable us to conduct more holistic studies integrating a much wider range of relevant parameters. Results from these studies will be crucial in the future to further our understanding of rhizosphere processes, as a few pioneer studies already showed that conclusions drawn from simplified systems might not hold true when studying complexes environments.

In summary, we suggest that including root traits and related belowground plant-soil-microbe interactions in our breeding efforts will help to select crops resilient to abiotic and biotic environmental stresses, like drought, flooding, poor nutrient availability, pest and pathogen attacks. In light of a growing world population and less predictable climatic conditions, we need to find solutions for crop production in a less resource-demanding manner which is also less detrimental to the environment. By focussing on a better understanding of individual plant traits, in conjunction with the associated microbiome and soil physicochemical properties as well as climatic conditions, we believe that it will be possible to further agroecological innovations in crop and soil management to increase soil health and reduce the footprint of agricultural practices.
